# Abdominal Cocoon and Adhesiolysis: A Case Report and a Literature Review

**DOI:** 10.1155/2013/381950

**Published:** 2013-02-17

**Authors:** Hassan Al-Thani, Jamila El Mabrok, Noof Al Shaibani, Ayman El-Menyar

**Affiliations:** ^1^Trauma Surgery Section, Department of Surgery, Hamad General Hospital, P.O. Box 3050, Doha, Qatar; ^2^Clinical Research, Trauma Surgery Section, Hamad General Hospital, Doha, Qatar; ^3^Clinical Medicine, Weill Cornell Medical College, P.O. Box 24144, Doha, Qatar

## Abstract

Abdominal cocoon is a rare cause of intestinal obstruction. It is difficult to diagnose in most of the cases preoperatively. Surgical removal of the membrane resulted in complete recovery in the majority of the cases. The exact etiology of abdominal cocoon is still unknown. We reported a male patient who presented with features of intestinal obstruction and has been diagnosed as abdominal cocoon intraoperatively.

## 1. Introduction

Abdominal cocoon is a rare condition that refers to total or partial encapsulation of the small bowel by a fibrocollagenous membrane or cocoon with local inflammatory infiltrate leading to acute or chronic bowel obstruction [1]. The condition has been described with various names including “peritonitis chronica fibrosa incapsulata” by Owtschinnikow in 1907 [2]. Abdominal cocoon is predominantly reported among females from the tropical and subtropical regions. However, adult males were also reported to represent cases of abdominal cocoon [1, 3–5]. Herein, we present a case with this condition that we believe to be the first one reported in Qatar.

## 2. Case Report

A 41-year-old male patient was admitted to the general surgery department of our hospital, complaining of abdominal pain, nausea, and vomiting. He had clinical history of several attacks of abdominal pain over the last seven months. He is a known case of eczema and was on local steroids since four years. The patient had no history of previous abdominal operation. On physical examination, a soft, nontender, and mobile mass was palpated in the right half of the abdomen. No hepatomegaly or splenomegaly was observed. Bowel sounds were a bit hyperactive, and rectal examination was normal. There was no abnormality in the complete blood count and blood chemistry.

**Figure 1:**
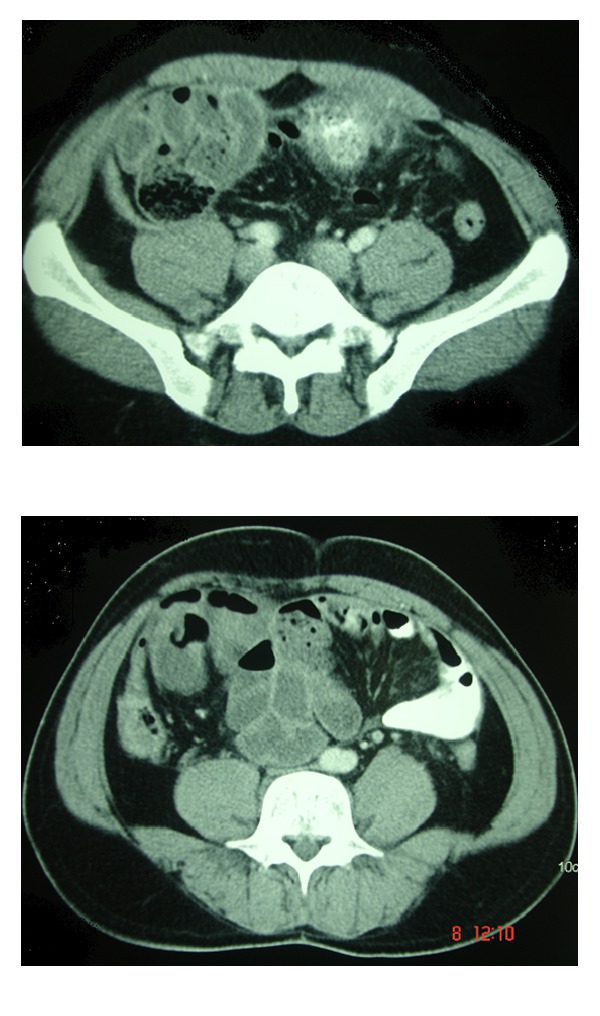
CT showing membrane enveloping loops of small bowel.

CT abdomen revealed multiple clumped small bowel loops in the lower abdomen on the right side with no passage of oral contrast. The adherent bowel loops showed wall enhancement with contrast (Figure 1).

**Figure 2:**
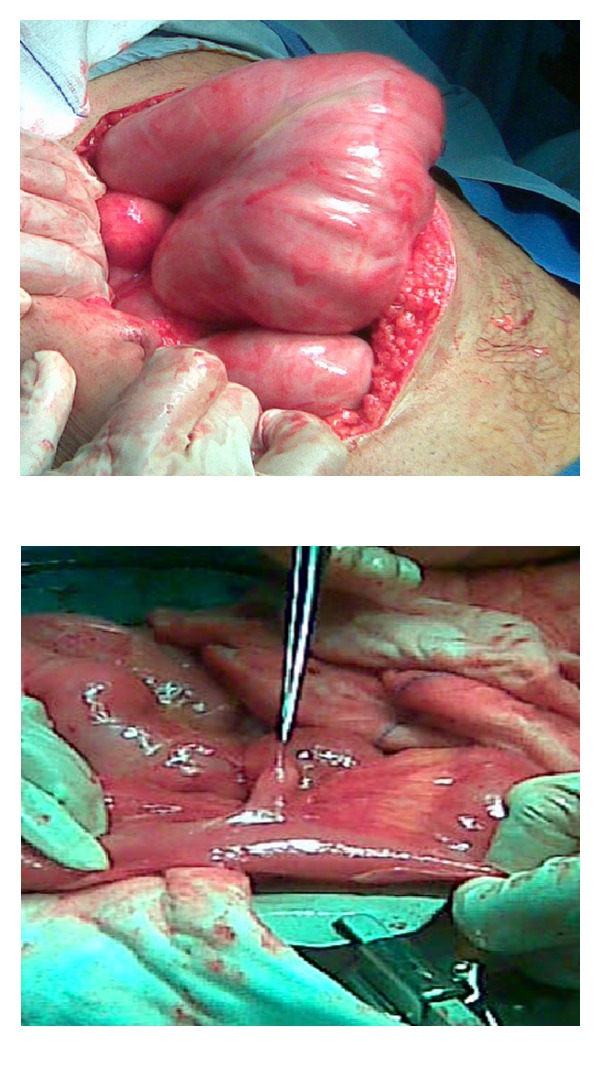
Intraoperative photograph shows the encapsulated small bowel with a dense fibrous layer.

The patient underwent emergency explorative laparotomy. The entire small bowel was found to be encased in a cocoon-like fibrous membrane which extended laterally to involve ascending and descending colon (Figure 2). A 2 cm diverticulum was seen in the terminal ileum. Other organs were normal. Lysis of the membrane was carried out, and loops were separated by dissection. The freed small bowel segments were viable, and excision of the diverticulum was done. 

Intraoperative findings showed encapsulation of small bowel by a dense whitish membrane as a cocoon, which was excised and adhesiolysis was done to release the loops of the intestine. The histological examination of the membrane revealed fibrous tissue focally lined by flatted mesothelial cells with scattered mononuclear inflammatory cell infiltrate and tissue culture fibroblasts. The patient showed a significant recovery postoperatively and was discharged from the hospital uneventfully.

## 3. Discussion

The preoperative diagnosis of abdominal cocoon is difficult and hence, the diagnosis is usually confirmed by laparotomy. The signs and symptoms of abdominal cocoon are usually nonspecific [6, 13, 14] including vomiting, abdominal pain and distention. The signs comprise soft abdomen with the presence of palpable nontender mass. In our case, the patient gave also history of recurrent episodes of abdominal pain over the last seven months, and he denied any history of previous abdominal operation.

Abdominal cocoon could be classified as primary (idiopathic) or secondary [14].  Table 1 shows a review of the literature for abdominal cocoon [3, 6–12]. 

The causative factor for primary form remains unknown, which might be caused by a subclinical peritonitis leading to the formation of a cocoon [15]. On the other hand, placement of LeVeen shunt for refractory ascites, continuous ambulatory peritoneal dialysis, tuberculosis, systemic lupus erythematosus, and the use of povidone iodine for abdominal washout as well as the beta-adrenergic blocker (practolol) are identified as secondary causes of abdominal cocoon [9]. Prevalence of sclerosing peritonitis among subjects under peritoneal dialysis is reported to be 0.7%, and the mortality rate varies from 56% to 93% despite various therapeutic modalities [11].

It is challenging to diagnose abdominal cocoon preoperatively, as these patients usually presented with normal biochemical investigations and imaging findings apart from CT and MRI are nonspecific [4]. Hence, often diagnosis is confirmed by laparotomy. It has been suggested that two clinical signs may be elicited. Firstly, in view of the dense fibrous sac, only bowel proximal to and out of the fibrous layer can distend and the patient may have a fixed, asymmetrical distension of the abdomen that does not vary with peristalsis. In addition, the patient would have a difference in consistency of the abdominal wall to palpation. This is because the distended area is soft and the flat area, which is covered by the dense fibrous capsule, is firm. In our patient, the entire small bowel was encased. Recurrent acute or chronic small bowel obstruction with increased compression of the encased intestine that causes abdominal pain has been reported in the majority of patients with abdominal cocoon syndrome [15, 16]. Clinical presentation also includes a palpable abdominal mass that resulted from encapsulated cluster of dilated small bowel loops. 

CT findings are consistent in diagnosing abdominal cocoon [16]. CT abdomen in our case also revealed multiple clumped small bowel loops in the lower abdomen and the adherent bowel loops showed wall enhancement with the use of contrast. However, intraoperative and histopathology findings are usually considered for final diagnosis of abdominal cocoon, as most patients underwent surgical intervention without imaging [17].

Encasement of the whole or part of the small bowel in a thick shiny membrane is a characteristic finding of abdominal cocoon [3]. In the present case, the entire small bowel was encased in a cocoon-like fibrous membrane which extended laterally to involve ascending and descending colon. Surgical management of abdominal cocoon is usually done by careful dissection and excision of the thick sac with the release of the small intestine. We also performed surgical removal of the membrane which leads to complete recovery. 

In conclusion, abdominal cocoon is a rare cause of small bowel obstruction. The preoperative diagnosis requires a high index of suspicion, supported by clinical data and imaging findings indicative of the condition. However, most cases are diagnosed at exploratory laparotomy. 

## Figures and Tables

**Table 1:** Review of the literature for abdominal cocoon.

Authors	Year	Age and gender	Country	Clinical association
Devay et al. [3]	2006	30-year male	Turkey	Acute appendicitis
She et al. [6]	2012	47-year woman	Hong Kong	Peritoneal dialysis
Sarmast et al. [7]	2012	30-year male	India	Tuberculosis
Serter et al. [8]	2012		Turkey	
Chatura and Nayak [9]	2012	14-year girl	Indian	Tuberculosis
Kayastha and Mirza [10]	2012	13-year girl	Pakistan	Suspected Acute appendicitis
Noormohamed and Kadi [11]	2012	N/A	UK	Peritoneal dialysis
Hur et al. [12]	2004	34-year woman 47-year man	South Korea	Unknown
This paper	2012	41-year male	Qatar	Unknown
